# A Systematic Review of Dietary Lifestyle Interventions for Neuropathic Pain

**DOI:** 10.3390/jcm13226766

**Published:** 2024-11-10

**Authors:** Michael Klowak, Rachel Lau, Mariyam N. Mohammed, Afia Birago, Bethel Samson, Layla Ahmed, Camille Renee, Milca Meconnen, Mahmud Sam, Andrea K. Boggild

**Affiliations:** 1 Institute of Medical Science, University of Toronto, 1 King’s College Circle, Medical Sciences Building, Room 2374, Toronto, ON M5S 1A8, Canada; 2 Public Health Ontario Laboratories, Public Health Ontario, 661 University Ave, Toronto, ON M5G 1M1, Canada; 3 Tropical Disease Unit, Toronto General Hospital, 200 Elizabeth Street, 13EN-218, Toronto, ON M5G 2C4, Canada; 4 Department of Medicine, University of Toronto, C. Davis Naylor Building, 6 Queens Pk Cres W 3rd Floor, Toronto, ON M5S 3H2, Canada

**Keywords:** lifestyle medicine, neuropathic pain, neuropathy, nutrition, systematic review

## Abstract

**Background/Objectives:** Chronic severe neuropathic pain (NP) affects one in 10 individuals over the age of 30 in North America. Standard pharmacological interventions are associated with significant side effects and have limited effectiveness. Diets seeking to improve physiological health, support gut barrier integrity, and decrease systemic inflammation have recently emerged as powerful tools conferring neuroprotective and anti-inflammatory effects, potentially reducing the overall morbidity and mortality of multiple neurological and metabolic diseases. This systematic review aimed to synthesize the literature around NP outcomes following dietary interventions compared to routine standard of care. **Methods:** Following PRISMA guidelines, an initial search yielded 15,387 records after deduplication. Six interventional trials specifically assessing dietary interventions for neuropathic pain were included and analyzed. The dietary lifestyle interventions included low-fat plant-based, plant-based fasting-mimicking, low-calorie, potassium-reduced, gluten-free, and intermittent high-protein/Mediterranean diets. **Results:** The included studies described some statistically significant improvements in pain severity on objective quantitative sensory testing, electrophysiology, imaging, and subjective questionnaires. The overall risk of bias was moderate, with only one trial demonstrating a low risk of bias across all assessed domains. No serious adverse events were identified, and dietary interventions were generally well tolerated. **Conclusions:** The data collected and synthesized in this systematic review indicate that dietary lifestyle interventions may offer a low-risk, low-cost, low-tech option for chronic neuropathic pain management, potentially improving quality of life and reducing overall morbidity. However, given substantial variability across studies and a moderate risk of bias, further research is warranted to substantiate these findings.

## 1. Introduction 

Chronic debilitating neuropathic pain (NP), requiring therapeutic intervention, affects up to 10% of the global population annually [1]. Several underlying etiologies, including physical injury, metabolic and autoimmune disorders, poor nutrition, harsh chemotherapeutics, infection, and genetics, contribute to the growing prevalence and incidence of NP [2,3,4,5]. A comprehensive systematic review assessing the global experience of NP suggests that post-herpetic neuralgia, trigeminal neuralgia, and painful peripheral neuropathy (PN) due to diabetes lead both prevalence and incidence (in person-years, PY) of NP at 0.07–0.09% (3.9–42/100,000 PY), 0.07% (12.6–28.9/100,000 PY), and 0.8% (15.3–72.3/100,000 PY), respectively [6]. Similarly, a comprehensive synthesis of NP in individuals with cancer suggests that up to 40% of cancer patients, including those receiving chemotherapeutics, have significant NP requiring additional treatment [7]. Therefore, as the prevalence and incidence of underlying etiologies continue to rise, effective gold-standard therapeutics to reduce the overall morbidity of NP are of increasing relevance.

Standard pharmacological interventions for NP are associated with significant side effects and limited effectiveness. First-line therapeutics such as tricyclic antidepressants, selective serotonin-norepinephrine reuptake inhibitors, and anticonvulsants including amitriptyline, duloxetine, gabapentin, and carbamazepine, are known to cause sedation, nausea, ataxia, and anticholinergic effects in up to 60% of individuals, often resulting in treatment interruption or cessation [8,9,10]. Similarly, second- and third-line therapeutics, including opioids such as morphine, oxycodone, and tramadol, are often avoided due to significant side effects and a high risk of addiction. Nausea, vomiting, constipation, lethargy, seizures, ataxia, and potential respiratory depression have been observed in up to 50% of patients receiving opioid treatment, resulting in statistically significant treatment cessation [11,12]. Regardless of the significant side-effect profiles of currently available NP therapeutics, patients who can tolerate treatment are frequently left with unsatisfactory results. Due to the limited efficacy of NP therapeutics, patients are at a greater risk for increased sleep disturbances, diminished quality of life, severe depression, and anxiety when left untreated [5,13,14,15,16]. In the absence of effective pharmaceuticals, alternative supportive interventions must be explored.

Lifestyle interventions have recently emerged as accessible and cost-effective strategies that may reduce the burden and severity of NP, particularly in type 2 diabetes mellitus (T2DM). Strategies seeking to improve physiological health, including those that reduce inflammation and enhance immune responsiveness to neurotoxins, are powerful tools that can influence underlying neuropathic etiologies. Proper management of lifestyle factors such as dietary patterns, physical activity levels, sleep quality, social connection, stress management, and avoidance of harmful substances such as tobacco and alcohol, may attenuate the formidable variables that exacerbate NP. Trials assessing lifestyle interventions have shown some statistically significant improvements in NP severity, therapeutic requirements, and overall quality of life [17,18,19,20,21].

Specifically, several studies have shown that diets promoting adequate vitamin and mineral intake enhance the analgesic effect of standard therapies, thereby alleviating symptoms associated with the inflammatory demyelinating processes underlying NP [22,23,24]. Plant-based, Mediterranean, and Nordic diets rich in whole grains, vegetables, legumes, nuts, seeds, fiber, and olive oil, influence pathogenesis of autoimmune, inflammatory, and metabolic conditions by conferring additional neuroprotective and anti-inflammatory effects, such as reducing oxidative stress and modulating neuro-inflammation. Diets seeking to improve physiological health, support gut barrier integrity, and decrease systemic inflammation have been effective in managing metabolic syndromes, ultimately reducing the overall morbidity of diabetic neuropathy and the mortality of coronary heart disease and cardiovascular disease [25,26,27,28,29,30]. This systematic review series aims to synthesize the literature surrounding lifestyle interventions for NP, such as diet, physical activity, health counselling, and mindfulness-based stress reduction. The first review in this series of systematic reviews focuses on dietary lifestyle interventions, which are hypothesized to improve the quality of life and reduce the burden of NP in affected patients.

## 2. Methods

### 2.1. Protocol and Registration

This systematic review was carried out according to the Preferred Reporting Items for Systematic Reviews and Meta-Analysis (PRISMA) guidelines [31] and was registered in the International Prospective Register of Systematic Reviews, PROSPERO (484158).

### 2.2. Eligibility Criteria

We included all studies that assessed lifestyle interventions and/or parameters, or that stratified outcomes by lifestyle factors, in patients with neuropathic pain due to any cause. Therefore, systematic reviews, randomized controlled trials (RCT), cohort studies, case-control studies, case-series, and case reports (n ≥ 1) were included. Lifestyle interventions and/or parameters involved dietary habits, exercise/physical activity levels, stress reduction, sleep quality, and avoidance of risky substances such as alcohol and tobacco. Participants in these studies comprised patients with various etiologies, including but not limited to diabetes, cancer, and alcohol use disorder, with no restrictions on age or sex. Interventional trials with gold-standard comparator groups as well as those with “no treatment” arms were included.

Studies with alternative methodological designs, such as non-human trials, trial descriptions/protocols, conference abstracts, editorials, and commentaries, were excluded. Articles assessing ocular neuropathies were also excluded due to the absence of objective, globally standardized reporting metrics and the potential increased risk of confounding from conditions such as retinopathy, cataracts, and non-ocular/eye nerve-related disorders.

### 2.3. Outcome Measures

Given the extensive breadth of tests used to assess NP, a wide range of reported outcomes have been collected when possible, including: 

(i) Efficacy: Subjective pain and neuropathy severity (on physical exam and questionnaires), and objective nerve function (via quantitative sensory testing (QST), electrophysiology, biopsy, imaging, and physical exam)

a. Questionnaires: Brief Pain Inventory (BPI) [32], European Organization for Research and Treatment of Cancer Quality of Life Questionnaire Core-30 (EORTC QLQ C-30) [33], Functional Assessment of Cancer Therapy—Neurotoxicity (FACT-NTX) [34], Gracely Pain Scale (GPS) [35], Leeds Assessment of Neuropathic Symptoms and Signs (LANSS) [36], McGill Pain Questionnaire (MPQ) [37], Michigan Diabetic Neuropathy Score (MDNS) [38], Michigan Neuropathy Screening Instrument (MNSI) [39], Neuropathic Pain Scale (NPS) [40], Neuropathy Quality of Life (NQOL) [41], Neuropathy Symptom Score (NSS) [42], Neuropathy Total Symptom Score (NTSS) [43], Numeric Pain Rating Scale (NPRS) [44], Present Pain Intensity (PPI) [45], Pain Severity Scale (PSS) [46], Patient Neurotoxicity Questionnaire (PNQ) [47], Short Form-36 Health Survey (SF36) [48], Subjective Peripheral Neuropathy Screening (SPNS) [49], modified Toronto Clinical Neuropathy Score (mTCNS) [50], Total Neuropathy Score (TNS) [51], Visual Analog Scale (VAS) [52].

b. QST: tuning fork vibration, mechanical pin prick, pressure, and temperature (warm, cold) pain detection, perception, and sensitivity thresholds

c. Electrophysiology: nerve conduction studies (conduction velocity, action potential amplitude, nerve excitability, and latency)

d. Biopsy with histopathological evidence of neuritis or neuropathy

e. Imaging: nerve fractional anisotropy, and t2-time

f. Physical exam: level of motor grade disability, abnormal peripheral nerve examination

(ii) Tolerability of lifestyle intervention (adherence vs. non-adherence and cessation)

(iii) Safety and adverse events (frequency and severity measures)

Demographic data, including age and sex, as well as trial setting, number of participants, underlying etiologies, and descriptions of relevant lifestyle interventions, were also collected, extracted, and reported.

### 2.4. Data Sources

Five electronic databases were searched, from database inception to August 2024, to identify relevant papers: Medline, PubMed, Scopus, Embase, and LILACS. No restrictions on language, publication status, or year of publication were implemented; however, the search strategy was restricted to humans. Bibliographies of relevant systematic reviews, reviews, trials, and key papers were hand-searched for additional literature.

### 2.5. Search Strategy

A comprehensive search strategy encompassing underlying neuropathic etiologies, lifestyle interventions, and stratifiers was implemented on all five databases:

(neuropathic pain OR neuropathy OR neuritis OR diabetic neuropathy OR peripheral neuropathy OR chemical neuropathy OR toxic neuropathy OR chemotherapy-induced peripheral neuropathy OR vitamin B deficiency) AND (nutrition OR nutrient OR nutritionally compromised OR micronutrient OR macronutrient OR malnutrition OR nutritional status OR nutrient supplement* OR plant based OR vegetarian OR vegan OR mediterranean diet OR diet OR physical activity OR exercise OR lifestyle OR lifestyle interventions OR BMI OR smoking OR alcohol OR stress OR sleep).

### 2.6. Study Selection

Titles and abstracts of all captured articles were assessed for inclusion and were discarded if they did not explicitly mention both lifestyle factors/interventions and neuropathic pain. The following inclusion criteria were followed for eligibility:

(i) any patient population irrespective of age or sex;

(ii) with neuropathic pain due to any cause (including non-specific neuropathy, neuritis, diabetic neuropathy, peripheral neuropathy, autonomic neuropathy, chemical neuropathy, toxic neuropathy, nutritional neuropathy, infectious neuropathy, and chemotherapy-induced peripheral neuropathy);

(iii) treated with or assessed for a lifestyle intervention or parameter (including nutritional interventions, malnutrition, plant-based diets, vegetarian diets, vegan diets, Mediterranean diets, other types of diet, physical activity, exercise, BMI, smoking, alcohol, stress, or sleep).

Document organization and deduplication, as well as title, abstract, and full-text screening was executed using the online platform Covidence. Articles were independently double screened by two reviewers and any discrepancies were resolved by a tertiary arbitrator.

### 2.7. Data Extraction

Data extraction was carried out by two independent reviewers and verified and collated by the study lead according to the Grading of Recommendations, Assessment, Development, and Evaluation (GRADE) framework [53,54]. Following extraction, “Summary of Findings” tables were generated using GRADEpro GDT (McMaster University, Hamilton, Canada). Non-English articles were screened and extracted by native-speaking reviewers or, in their absence, were translated into English using Google translate (Google, Mountain View, CA, USA). All discrepancies were resolved through discussion among multiple reviewers.

### 2.8. Statistical Analysis

Continuous variables from questionnaires and nerve conduction studies were collected and reported as sample sizes, means, mean differences, standard deviations, medians, and interquartile ranges where applicable. Dichotomous or categorical variables (e.g., presence of neuropathy, pain severity, etc.) were collected and reported as frequencies and proportions, with 95% confidence intervals when provided. Continuous outcomes (mean difference) and dichotomous outcomes (relative risk and odds ratio) were collected when available, and reported in the summary of findings tables, only when the primary study included a comparator group, using a standardized measure of treatment difference. Summary estimates of both continuous and dichotomous outcomes were pooled using a parametric random effects model and inversely weighted by the sample size of primary studies for each combination of lifestyle intervention and neuropathic outcome. Level of significance was set at a 5% alpha level for summary estimates of outcomes measured against a comparator. Statistical analysis was carried out using GRADEpro GDT (McMaster University, Hamilton, Canada) and Review Manager (RevMan, computer program, version 5.3. Copenhagen: The Nordic Cochrane Centre, The Cochrane Collaboration, 2014).

### 2.9. Risk of Bias and Certainty of Evidence

Comprehensive risk of bias (ROB) forms, adapted from the Joanna Briggs critical appraisal tools, were designed and subsequently utilized independently and simultaneously by two reviewers to carry out the bias assessment [55]. The GRADE framework was followed to assess methodological quality, assigning each included study a quality grade of high, moderate, low, or very low, based on apparent level of bias [53,54]. Discrepancies were resolved by a tertiary arbitrator through discussion.

The GRADE framework is a common and transparent approach to grading certainty or “quality” of evidence and the strength of recommendations. At baseline, RCTs are given high GRADE scores, while observational studies are deemed to have low GRADE scores [53,54]. These scores may then be demoted due to apparent bias variables, such as selection bias, detection bias, attrition bias, reporting bias, and information/outcome bias, or promoted based on the relative strength of the association, presence of a dose-response gradient, and potential confounding factors [53,54]. Despite the systematic nature of the GRADE framework, subjective judgements based on the perspectives of the reviewers and context of the review remain relevant and necessary. This consideration is of particular relevance to studies assessing lifestyle interventions, where some forms of bias are intrinsic. Therefore, rather than utilizing ROB platforms designed for lifestyle medicine that leave less room for subjectivity, we chose to apply the GRADE framework.

The specific methodology adhered to by reviewers for assessing potential bias in RCTs is as follows. Studies were demoted if selection bias was apparent on the grounds of inadequate allocation. Truly random allocation processes, such as software-based random number generation, third-party centralized randomization, and concealment via sealed envelopes, were sufficient, whereas a non-random process such as utilizing personal information (for example, date of birth) was considered a potential ROB. As many lifestyle interventions cannot be realistically double blinded, given subjects active participation in a specific diet or exercise, detection bias was only assessed based on the blinding of study personnel collecting data. Inadequacy of such blinding resulted in potential detection bias. The apparent level of participant loss across study groups was also assessed for potential attrition bias. A > 10% loss within a single group, relevant imbalance between groups due to withdrawals, or failure to report loss, potentially effecting outcomes, was considered a potential source of bias. Lastly, completeness of reporting (reporting bias) and the objectivity of the outcomes (information/outcomes bias) was also assessed. If all outcomes were not reported or were incompletely reported, or if the assessments carried a level of subjectivity (such as questionnaires), studies were considered at risk for reporting and/or information/outcome bias, respectively. Bias assessments were pooled and an overall ROB score was achieved per study.

Additional GRADE parameters such as inconsistency, indirectness, and imprecision of outcomes, as well as apparent levels of publication bias and plausible confounding, effect size, and relevant dose response gradients were also considered when grading certainty of evidence. Certainty was upgraded by one additional unit if a large effect size (<0.5 or >2) was evident, or by two additional units if a very large effect size (<0.2 or >5) was reported. Overall, ROB was then considered alongside these additional GRADE parameters to generate a final certainty of evidence GRADE score, per reported outcome.

## 3. Results

### 3.1. Literature Search

**Figure 1:**
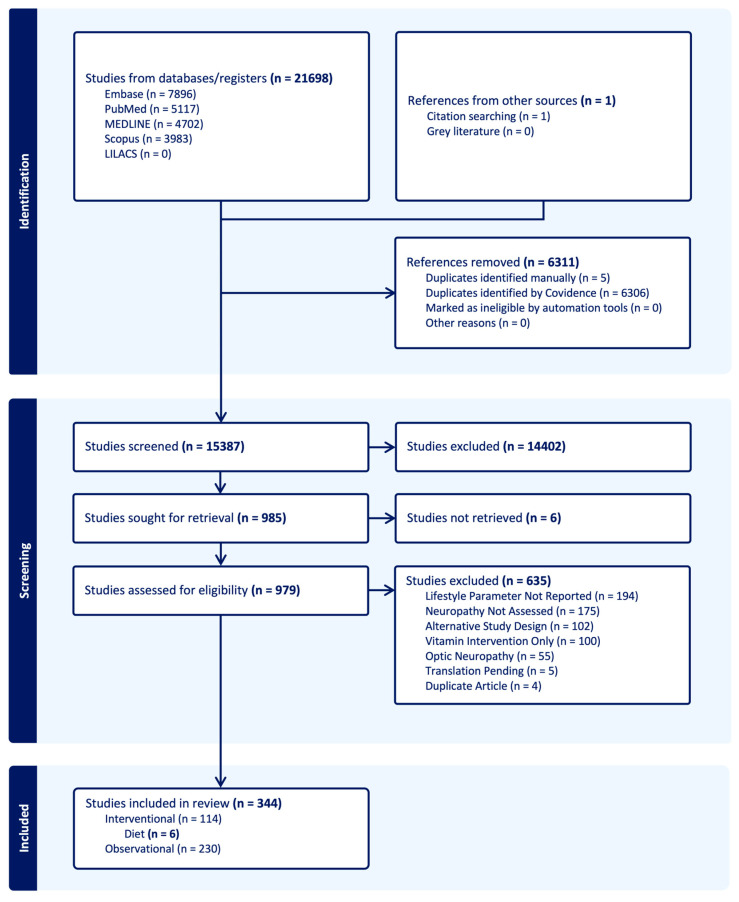
PRISMA flowchart.

A total of 21,698 articles were identified, using the search strategy, from Embase (7896), PubMed (5117), Medline (4702), Scopus (3983), LILACS (0), and additional citations from bibliographies (1). Of the 15,387 articles that were identified after deduplication, 985 full-text articles were collected and screened for final inclusion. A total of 344 articles fulfilled inclusion criteria for qualitative synthesis, including 230 observational studies and 114 interventional studies. Articles were primarily excluded because they either did not report a lifestyle intervention or parameter (194), or did not report neuropathic outcomes (175). A full list of article disposition is available in Figure 1 and Supplementary Table S1. Six interventional trials assessing the role of specific dietary interventions on peripheral sensory and/or motor neuropathies are reported herein, and characteristics of each study are documented in Table 1 [18,56,57,58,59,60].

### 3.2. Included Studies

**Figure 2:**
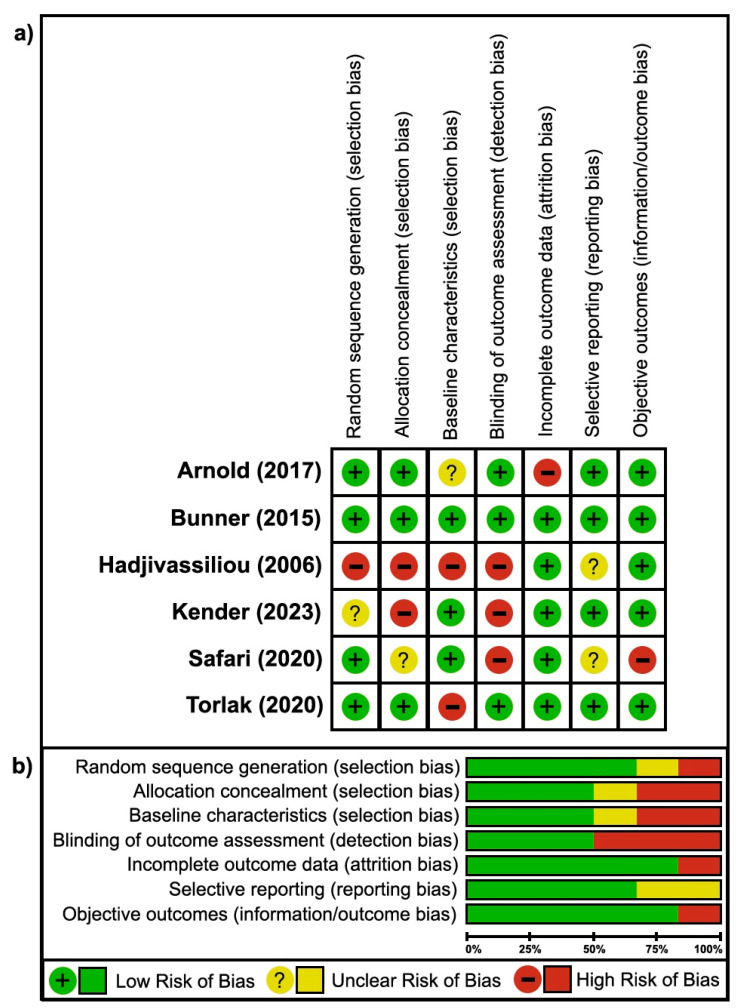
Risk of bias assessment for interventional trials. (**a**) Risk of bias summary by randomized control trial; (**b**) summary of risk of bias items by bias item [18,56,57,58,59,60].

Almost all trials were conducted in high-income countries (5/6), followed by low-middle-income countries (1/6) as defined by the World Bank from 1979 to 2019 (Table 1) [61]. Dietary lifestyle interventions included a low-fat plant-based diet (1/6) [18], plant-based fasting-mimicking diet (1/6) [58], low-calorie diet (1/6) [60], potassium-reduced diet (1/6) [56], gluten-free diet (1/6) [57], and an intermittent high protein/Mediterranean diet (1/6) [59], each with specific guidelines and varying durations of implementation. All trials (6/6) had at least a 2-arm study design, comparing the efficacy of lifestyle interventions against control-routine standard of care (Table 1). Likewise, all trials evaluated individuals with neuropathy or NP; however, the underlying etiologies varied per study. The majority of trials assessed a cohort of individuals with type 2 diabetes mellitus (2/6) [18,58], followed by stage 3/4 chronic kidney disease (1/6) [56], chronic sciatica (1/6) [60], chronic lower back pain (1/6) [59], and gluten sensitivity (1/6) [57]. Trials reported outcomes using comprehensive questionnaires (5/6) [18,56,58,59,60], electrophysiology (4/6) [18,56,57,58], QST (4/6) [18,56,58,60], and imaging (1/6) [58] (Table 1). Lastly, the ROB assessment for all studies is summarized in Figure 2 [18,56,57,58,59,60].

Six trials assessed NP outcomes following dietary interventions, compared to routine standard of care (Table 1). A comprehensive RCT, conducted by Bunner and colleagues (2015), assessing a low-fat plant-based diet plus vitamin B12 (1000 mcg/day) supplementation in individuals with T2DM and PN, showed a statistically significant improvement on comprehensive questionnaires [18]. The low-fat plant-based diet intervention improved neuropathy and NP outcomes, including the change in MPQ, MNSI, and NTSS within the intervention group (*p* < 0.01 for all). However, a significant improvement in foot conductance, VAS, and NTSS was also described in the control group receiving standard of care (*p* < 0.05, *p* < 0.05, *p* < 0.01, respectively). Between-group analysis also suggested an improvement in NP by the change in MPQ, MNSI, and foot conductance (*p* = 0.04, *p* = 0.03, *p* = 0.03, respectively). Additional metrics, including MNSI-PA, VAS, NTSS, and hand conductance remained unremarkable between groups. The methodological quality of the study was high, and no adverse events were reported alongside a >75% adherence to the lifestyle intervention [18] (Table 1, Table 2 and Table S2, Figure 2).

In a similar RCT assessing a plant-based fasting-mimicking diet in participants with T2DM, statistically significant improvements in NP outcomes were infrequently observed [58]. Kender and colleagues (2023) reported that the plant-based fasting-mimicking dietary intervention improved tibial nerve compound muscle action potentials within the intervention group (*p* < 0.05); however, improvements in tibial motor nerve conduction velocity (NCV) and heat pain threshold were also described within the control group (*p* < 0.05 each) receiving a Mediterranean diet. Remaining QST and electrophysiological outcomes within and between groups were non-significant, and the methodological quality of evidence of this paper was low. Adverse events were not specified, and there was high adherence to the intervention with no loss to follow-up reported [58] (Table 1, Table 2 and Table S2, Figure 2).

In an RCT assessing a low-calorie diet (1200 kcal/day) in patients with chronic sciatica and NP [60], Safari and co-authors (2020) described statistically significant improvements in pain questionnaires. The low-calorie dietary intervention resulted in improved MPQ sensory (*p* < 0.001), affective (*p* = 0.002), total (*p* < 0.001), and PPI (*p* = 0.001) scores. Although PPI also increased in the control group receiving standard of care (*p* = 0.013), MPQ sensory (*p* = 0.015), affective (*p* = 0.002), total (*p* = 0.001), and PPI (*p* = 0.006) were significantly improved during between-group analysis adjusted for baseline. A high level of adherence was described with no loss to follow-up; however, safety and tolerability data were not reported, and the methodological quality of evidence was low [60] (Table 1, Table 2 and Table S2, Figure 2).

Likewise, in an RCT assessing a potassium-reduced diet (1 mmol/kg/day) in patients with stage 3 or 4 chronic kidney disease [56], Arnold and colleagues (2017) described a statistically significant improvement in neuropathy as assessed by change in the TNS (*p* < 0.01), composite nerve excitability score (*p* = 0.04), and gait speed (*p* = 0.01) between groups. Intragroup analysis of questionnaires and electrophysiology was unremarkable. In this RCT, no adverse events were reported, 8.7% and 12.5% loss to follow-up rates were observed in the intervention and control groups, respectively, and the methodological quality of evidence was moderate overall [56] (Table 1, Table 2 and Table S2, Figure 2).

In a trial conducted by Hadjivassiliou and colleagues (2006) assessing dietician-led gluten-free dietary counselling for participants with gluten sensitivity-related neuropathy [57], a statistically significant improvement in the change in sural sensory nerve action potential was found within the interventional group, control group, and between groups (*p* < 0.001, *p* < 0.01, and *p* < 0.03, respectively). Within- and between-group analyses of sural NCV was non-significant. Additionally, a gluten-free diet resulted in a subjective improvement in neuropathy perception in 64% of participants, while 80% of participants in the control group described noticeable deterioration. A high degree of dietary adherence was described; however, methodological quality was low, and safety or tolerability data were not mentioned [57] (Table 1, Table 2 and Table S2, Figure 2).

Lastly, Torlak and colleagues (2020) found that an intermittent high protein/Mediterranean diet also resulted in significant improvements in NP on VAS and LANSS within all study groups experiencing chronic lower back pain (*p* < 0.001 throughout) [59]. Despite this, between group analyses remained non-significant for both outcomes (*p* = 0.111 and *p* = 0.134, respectively). The methodological quality of this study was moderate; however, there were no adverse events specified, 100% adherence, and no loss to follow-up reported [59] (Table 1, Table 2 and Table S2, Figure 2).

### 3.3. Risk of Bias

The overall ROB for all RCTs was moderate, as risk was deemed low in 64% (27/42) of all measures (Figure 2). The most common sources of bias were detection and selection biases, as half of all RCTs failed to blind study personnel to outcome determination (3/6, 50%) [57,58,60], properly conceal group allocation (3/6, 50%) [57,58,60], and reported limited characteristic matching across study groups (3/6, 50%) [56,57,59]. Selection bias was, however, bolstered by proper random sequence generation, achieved in 66% (4/6) [18,56,59,60] of all RCTs. Additionally, there was an overall low ROB in both reporting and information/outcome bias, as RCTs commonly reported all outcomes (4/6, 66%) [18,56,58,59], utilizing objective measures 83% (5/6) [18,56,57,58,59] of the time. Likewise, there was a low risk of attrition bias, as almost all RCTs described low loss to follow-up rates (5/6, 83%) [18,57,58,59,60]. Only one RCT achieved a low ROB across all items [18], as most (4/6, 66%) [56,57,58,60] described an unclear or high ROB from two or more potential sources [56,57,58,59,60] (Figure 2).

## 4. Discussion

### 4.1. Summary of Findings

Quality evidence supporting lifestyle interventions in the treatment of NP due to several underlying etiologies is limited. Current RCT-level data suggests that dietary lifestyle interventions have the potential to reduce the subjective and objective burden of NP in a variety of affected patient populations. However, there is a significant lack of large, comprehensive, high-quality trials assessing dietary lifestyle interventions within specific populations reporting the same outcomes. Consequently, given the wide breadth of assessments used in the diagnosis of NP, meta-analysis was not possible. Likewise, the grading of recommendations was mostly limited to ROB alone, with some outcomes upgraded given a large effect size. Overall, dietary-based lifestyle interventions did not significantly increase the incidence of serious adverse events across all trials and were generally well tolerated, reporting a few beneficial effects for NP [18,56,57,58,59,60] (Table 1, Table 2 and Table S2, Figure 2).

#### 4.1.1. Summary of Findings-Efficacy

Six trials assessing individual dietary lifestyle interventions were identified in this systematic review, each with highly specific outcomes, efficacy levels, and methodological certainty and quality of evidence. Bunner and colleagues (2015) reported a statistically significant improvement in NP on multiple questionnaires, including MNSI, MPQ, and NTSS, in an interventional trial assessing the implications of a low-fat plant-based diet in individuals with T2DM [18]. Marked improvements in glucose control, hypertension, dyslipidemia, and obesity were also reported, coinciding with the previous established literature. Therefore, it is highly probable that the neuroprotective effects in those with T2DM in this trial were underpinned by the improvements in glycemic control, blood pressure, lipid profiles, and BMI achieved during the trial. A large effect size was identified in the trial, strengthened by comprehensive methodology achieving a high certainty of evidence overall. Although the effect cannot be specifically attributed to one variable alone, the efficacy of a low-fat plant-based diet in the treatment of severe NP remains evident [18].

In a similar trial examining a plant-based fasting-mimicking diet with a Mediterranean diet, statistically significant improvements in NP outcomes were infrequently observed across various QST and electrophysiological assessments [59]. Statistically significant results were observed in both RCT groups irrespective of the intervention, and between-group analysis was unremarkable [58]. Although these trials may not be directly compared given the significant differences in study design, noteworthy assumptions may be extrapolated from their respective outcomes alone. Discrepancies between these studies may be attributable to the intermittent nature of the trial conducted by Kender and colleagues (2023) [58]. Additionally, although both described statistically significant reductions in weight loss, only the trial conducted by Bunner (2015) and colleagues described significant improvements in NP. This may suggest that a plant-based diet confers neuroprotective effects irrespective of the degree of associated weight loss; however, more robust and mechanistic data are needed to confirm this hypothesis. Regardless of this potential relationship, the Kender (2023) trial suffered from significant selection and detection biases, resulting in a low methodological quality of evidence overall. As a result, and although the current literature supports that intermittent fasting positively influences diverse neurological disorders such as epilepsy and multiple sclerosis, via cellular signaling pathways implicated in neuronal function and regulation of pain perception (including but not limited to signal-regulated kinases, rapamycin signaling, and opioid receptor expression pathways), the efficacy of an intermittent plant-based fasting-mimicking diet for the treatment of NP cannot be directly ascertained, and additional data are required [58,62,63,64,65].

Comparably, a low-calorie diet also resulted in a statistically significant reduction of NP on comprehensive questionnaires. Intergroup analysis of the MPQ and PPI suggests that a low-calorie diet reduces NP associated with chronic sciatica when compared to standard diets. Although weight loss also remained significant between groups, the authors suggest that changes in weight were clinically insignificant, and that improvement in NP morbidity may be the result of alleviating underlying inflammation, irrespective of the degree of the associated weight loss. Although significant detection and information/outcome bias reduced the methodological certainty of evidence of this trial to moderate, a low-calorie diet may confer positive beneficial effects on NP morbidity to some degree [60]. These findings coincide with the current body of literature, which suggests that a low-calorie diet reduces oxidative stress, modulates neurotrophic factors, and improves mitochondrial function, thereby reducing neuroinflammation and improving neuronal function overall [66,67].

In a trial assessing a potassium-reduced diet in individuals with stage 3/4 chronic kidney disease, the severity of NP was significantly reduced on TNS and electrophysiology [56]. The authors suggest that lowering potassium consumption resulted in enhanced control of nerve and muscle ion channel regulation, thereby reducing NP severity overall. This notion is underpinned by the previous literature demonstrating that high potassium levels are a significant risk factor to the development of uraemic neuropathy in those with chronic kidney disease [68,69]. Although a high rate of attrition was apparent in this trial, the strength of evidence remains high due to an overall low ROB on all other items, suggesting that a potassium-reduced diet may be efficacious in reducing NP morbidity [56].

Lastly, the efficacy of a gluten-free diet [57] and an intermittent high-protein/Mediterranean diet [59] examined in the remaining RCTs remains questionable. Although a gluten-free diet resulted in a statistically significant improvement in electrophysiological measures, the strength of evidence was severely diminished by a significant amount of both selection and detection biases. Conversely, although Torlak and colleagues (2020) demonstrated a low risk of bias overall, and a high methodological quality of evidence, a statistically significant improvement of NP between groups was not identified. The existing literature, on which the biological plausibility of high-protein and Mediterranean diets were predicated, supports that NP might be theoretically mitigated owing to substantial and maintained weight loss, suppression of pro-inflammatory markers, and increased neurotrophic factors and brain plasticity, which ultimately reduce inflammation and ameliorate neuropathic symptoms [70,71,72]. Likewise, although similar neuroprotective effects may be conferred by a gluten-free diet [73], findings may not be as broadly applicable, given that the intervention was primarily selected based on a patient population experiencing significant gluten sensitivity. As such, and to fully interrogate the potential contributions of gluten-free, high-protein, and Mediterranean diets on NP, more robust trials are required in order to ascertain the efficacy of these respective interventions [57,59].

Overall, dietary lifestyle interventions did exhibit some beneficial effects on NP severity in a variety of populations. Specifically, a low-fat plant-based diet, low-calorie diet, and a potassium-reduced diet may confer positive beneficial neuroprotective and anti-inflammatory effects that reduce NP severity overall [18,56,60]. In the case of the potassium-reduced diet, it is important to recognize that, while such an intervention may be appropriate for a patient population affected by chronic kidney disease, such an intervention in those with normal renal function may be deleterious. As such, the findings of that particular trial are unlikely to generalize as broadly as those of the assessed interventions that would be safe across large populations of patients with NP, regardless of etiology (e.g., a whole-food, plant-based diet) [74]. Additionally, given the plentiful and complex pathways that diet and dietary constituents can influence, it is difficult in such trials to disentangle the causal relationships hypothesized in the available literature. Therefore, more comprehensive interventional trials assessing the specific effects of dietary interventions on NP severity, as well as sophisticated mechanistic studies, are required to estimate the overall strength of evidence and nature of this relationship.

#### 4.1.2. Summary of Findings: Safety and Tolerability

Simple, cost-effective strategies to optimize physiological health in patients suffering from chronic disease have the potential to avoid the significant side effects and limited effectiveness of gold-standard pharmacologic therapeutics for NP. Low-risk lifestyle interventions, such as modifying one’s diet, have been shown to reduce NP severity with a relatively inconsequential side-effect profile. In this synthesis, we have demonstrated no significant adverse events of any kind when NP, due to a variety of underlying causes, was treated with dietary lifestyle interventions versus standard care. However, in the case of the potassium-reduced dietary intervention trial, the patient population was known to have moderate to severe chronic kidney disease. Beyond the effects commonly associated with standard of care, no other minor or major adverse events were attributed to any dietary lifestyle intervention across all synthesized trials. Overall, our review demonstrated that dietary lifestyle interventions improved NP severity without causing significant harm to study participants. Although subjective tolerability was not directly conveyed, almost all included studies reported >75% adherence and limited drop-out rates, suggesting that these lifestyle interventions were well tolerated [18,56,57,58,59,60]. The literature synthesized in this systematic review suggests that dietary lifestyle interventions known to be physiologically beneficial have the potential to improve NP severity while avoiding the significant side effects associated with gold-standard therapeutics.

### 4.2. Limitations

Although six interventional trials were identified in this systematic review, each examined a different dietary lifestyle intervention. Additionally, the assessments implemented to identify NP severity varied significantly across all studies [18,56,57,58,59,60]. Consequently, based on variation in study interventions, underlying etiologies, and measured outcomes, it was not possible to perform a meta-analysis. Likewise, between-study analysis of GRADE variables such as outcome inconsistency, indirectness, and imprecision could not be conducted. This systematic review is therefore limited by an abridged GRADE portfolio, as the certainty of evidence was largely based on the ROB assessment. Furthermore, the overall quality of evidence was moderate at best, as many interventional trials suffered from significant selection and detection biases. Although the included studies encompassed the global experience of chronic NP and were, in some cases, generalizable to a larger population, cohort sizes of individual trials remained relatively small. In addition, some trials tailored their dietary interventions to the specific underlying etiologies of their NP cohort, such as chronic kidney disease or gluten sensitivity, which limits the applicability of these findings across geographies or patient populations. Finally, while almost all included studies reported a statistically significant improvement in NP severity due to dietary lifestyle interventions, many markers of NP were not reported [18,56,57,58,59,60]. Therefore, although the data collected in this systematic review suggest that dietary lifestyle interventions are efficacious in the treatment of NP severity, larger and more robust interventional trials assessing standardized outcomes of NP are required to establish novel clinical recommendations according to causative etiology. However, the reported data certainly underscore the potential benefits of both plant-based Mediterranean-style and low-calorie diets for individuals suffering from the common form of NP in T2DM.

## 5. Conclusions

This systematic review synthesizes the current literature on dietary lifestyle interventions for the treatment of chronic and severe NP caused by various etiologies. Our findings align with the current literature suggesting that dietary lifestyle interventions known to be physiologically beneficial [74] may improve the quality of life and reduce the burden of severe NP in patients, particularly in those with NP due to T2DM. Specific diets, such as low-fat plant-based and low-calorie diets, may confer neuroprotective and anti-inflammatory effects [74], having briefly shown positive beneficial effects on NP severity via objective QST, electrophysiology, and subjective questionnaires. Similarly, in those with chronic kidney disease, a potassium-reduced diet may have similar effects. Moreover, the synthesized data consistently support a limited side-effect profile of such dietary interventions with few or no adverse events and excellent tolerability, a vast improvement over gold-standard therapeutics [18,56,60]. This systematic review has also identified gaps in the literature requiring further examination. Insufficient data were available for meta-analysis, largely due to the wide breadth of diagnostic tests available to document NP severity. Additionally, noteworthy sources of bias were detected in the included studies, resulting in a moderate certainty of evidence overall. In summary, dietary lifestyle interventions have been shown to be low-risk, low-cost, low-tech adjunctive therapies for chronic NP, particularly in those with T2DM. However, large-scale and comprehensive interventional trials reporting specific NP outcomes are warranted to strengthen the current evidence base. Further recommendations for dietary lifestyle-based NP therapeutics will be predicated upon the availability of further primary studies providing novel data sets, as well as updated syntheses.

## Figures and Tables

**Table 1:** Characteristics of six interventional trials included in this study.

Author (Year)	Setting	N	Mean Age (SD)	Range	Sex N (F:M)	Population/Etiology	Lifestyle	Outcomes (Mean ± SD)
Bunner (2015) [18]	US	34	Int: 57 (6); Con: 58 (6)		Int: 8:9; Con: 11:6	T2DM + PN	Low-fat plant-based diet + 1000 mcg vitamin B12/day ^Ψ^ for 5 months	**Efficacy:** Improvement of pain on MPQ (22.6 ± 11 vs. 13.5 ± 10 **), MNSI (7.5 ± 2.5 vs. 5.3 ± 2.5 **), and NTSS (10.7 ± 4.9 vs. 6.8 ± 4.5 **) within int. group, and in the change in MPQ (−9.1 ± 11.4 vs. −0.9 ± 11.3 *), MNSI (−2.2 ± 2.4 vs. −0.6 ± 1.5 *), and feet conductance (0.7 ± 10.5 vs. −11.7 ± 13.2 *) between groups
								**Safety:** No AE observed.
								**Tolerability:** ~76% adherence.
Kender (2023) [58]	Germany	31	Int: 66.6 (5.8); Con: 67.1 (5.9)	50–75	Int: 5:12; Con: 5:9	T2DM	Plant-based fasting-mimicking diet for 1 week/month for 6 months	**Efficacy:** Improvement in tibial motor nerve conduction velocity (37.23 ± 2.38 vs. 32.89 ± 3.05 *), and HPT (−0.76 ± 0.37 vs. −1.10 ± 0.30 *) within con. group, and tibial nerve compound muscle action potential (7.79 ± 1.24 vs. 9.21 ± 1.45 *) within int. group
								**Safety:** Mentioned “low” but AEs not specified
								**Tolerability:** High adherence and no L2FU
Safari (2020) [60]	Iran	96	Int: 39.67 (10.66); Con: 40.21 (10.46)	Int: 26–59; Con: 24–60	Int: 20:28; Con: 21:27	Chronic Sciatica + NP	Low calorie diet for 30 days	**Efficacy:** Improvement in MPQ sensory (6.73 ± 1.41 vs. 4.46 ± 1.71 ***), affective (0.98 ± 0.64 vs. 0.50 ± 0.62 **), total (7.71 ± 1.69 vs. 4.96 ± 2.02 ***) scores, and PPI (2.23 ± 0.47 vs. 2 ± 0.68 ***) within int. group, PPI (2 ± 0.68 vs. 1.79 ± 1.3 *) within con. group, and MPQ sensory (4.46 ± 1.71 vs. 5.74 ± 2.11 *), affective (0.50 ± 0.62 vs. 0.87 ± 0.85 **), total (4.96 ± 2.02 vs. 6.62 ± 2.53 ***) scores, and PPI (1.02 ± 0.98 vs. 1.79 ± 1.3 **) between groups adjusted for baseline
								**Safety:** Not mentioned
								**Tolerability:** 100% adherence and no L2FU
Arnold (2017) [56]	Australia	47	Int: 67; Con: 66	52–69	Int: 10:13; Con: 7:17	Stage 3/4 Chronic Kidney Disease	Potassium- reduced diet (1 mmol/kg/day) ^§^ for 2 years	**Efficacy:** Improvement in the change in TNS (0.4 ± 2.2 vs. 2.8 ± 3.3 **) and nerve excitability score (5.1 ± 2.8 vs. −2.3 ± 2.2 *) between groups
								**Safety:** No AE observed.
								**Tolerability:** 8.7% L2FU in int. group & 12.5% L2FU in con. group.
Hadjivassiliou (2006) [57]	UK	35	Int: 67.2 (2); Con: 70.9 (1.9)			Gluten Sensitivity ^†^ + PN	Gluten-free diet including counselling from expert dietician for 1 year	**Efficacy:** Improvement in the change in sural sensory nerve action potential amplitude within the int. group (1.39 ± 0.22 vs. 2.15 ± 0.43 ***), con. group (1.39 ± 0.47 vs. 0.96 ± 0.29 **), and between groups (0.76 ± 0.31 vs. −0.42 ± 0.25 *)
								**Safety:** Not mentioned.
								**Tolerability:** High adherence.
Torlak (2020) [59]	Turkey	60	Diet Group: 50.3 (1.64); Diet + PT Group: 54.30 (1.38); PT Group: 54.85 (3.81)		Diet Group: 10:10; Diet + PT Group: 10:10; PT Group: 10:10	Chronic Lower Back Pain + NP	Intermittent high protein diet (2 days/week) and Mediterranean diet (5 days/week) for 5 weeks	**Efficacy:** Improvement in VAS (8.3 ± 0.36 vs. 4.7 ± 0.41 ***; 7.45 ± 0.44 vs. 4.7 ± 0.42 ***; 6.65 ± 0.31 vs. 3.1 ± 0.59 ***) and LANSS (4.8 ± 0.88 vs. 2.3 ± 0.59 ***; 10.6 ± 0.88 vs. 7.1 ± 0.76 ***; 5.1 ± 0.42 vs. 2.6 ± 0.36 ***) within diet group, diet + PT group, and PT group, respectively
								**Safety:** Mentioned “low” but AEs not specified
								**Tolerability:** 100% adherence and no L2FU

AE: adverse events; BMI: body mass index; Con: Control; DM: diabetes mellitus; HPT: heat pain threshold; Int: intervention; L2FU: loss to follow-up; LANSS: Leeds assessment of neuropathic symptoms and signs; MNSI: Michigan neuropathy screening instrument questionnaire; MPQ: McGill pain questionnaire; NP: neuropathic pain; NTSS: neuropathy total symptom score; PN: peripheral neuropathy; PPI: present pain intensity; PT: physical therapy; T2DM: type 2 diabetes mellitus; TNS: total neuropathy score; VAS: visual analog scale. Outcomes reported as “between groups” compare the intervention group to the control group, while “within group”outcomes compare pre- intervention to post-intervention; *p* < 0.05: *; *p* < 0.01: **; *p* < 0.001: ***; ^†^ with 28% of participants demonstrating histopathological evidence of gluten enteropathy; ^§^ including: energy (if BMI > 30), sodium (<100 mmol/day), and phosphate (<1000 mg/day) restriction; ^Ψ^ including: omitting animal products, limiting fat intake to 20–30 g/day, and favouring low-glycemic index foods.

**Table 2:** Summary of findings on dietary lifestyle interventions compared to routine standard of care to manage symptoms of neuropathic pain.

Patient or Population: Patients with T2DM and Peripheral Neuropathy Setting: High-Income Countries (United States) (Bunner et al., 2015) [18] Intervention: Low-Fat Plant-Based diet, Plus Vitamin B12 (1000 mcg/Day) Supplementation Comparison: Standard Care					
Outcomes	Anticipated Absolute Effects *		№ of Participants (Studies)	Certainty of Evidence (GRADE)	Comments
	Risk with Standard Care	Risk with Diet (95% CI)			
MPQ-SF	The mean change in MPQ-SF was **0**	MD **8.2 lower** (−15.83, −0.57)	34 (1 study)	⨁⨁⨁⨁ High	Dietary lifestyle intervention reduced pain severity.
VAS	The mean change in VAS was **0**	MD **0.8 higher** (−1.15, 2.75)	34 (1 study)	⨁⨁⨁⨁ High	No difference in VAS.
MNSI-Q	The mean change in MNSI-Q was **0**	MD **1.6 lower** (−2.95, −0.25)	34 (1 study)	⨁⨁⨁⨁ High	Dietary lifestyle intervention reduced neuropathy severity.
MNSI-PA	The mean change in MNSI-PA was **0**	MD **0.3 higher** (−0.91, 1.51)	34 (1 study)	⨁⨁⨁⨁ High	No difference in MNSI-PA.
NTSS	The mean change in NTSS was **0**	MD **0.7 lower** (−3.33, 1.93)	34 (1 study)	⨁⨁⨁⨁ High	No difference in NTSS.
Feet Conductance (uS)	The mean change in feet conductance (uS) was **0**	MD **12.4 higher** (1.95, 22.85)	10 (1 study)	⨁⨁⨁⨁ High	Dietary lifestyle intervention improved foot conductance.
Hands Conductance (uS)	The mean change in hands conductance (uS) was **0**	MD **8.9 higher** (−2.36, 20.16)	10 (1 study)	⨁⨁⨁⨁ High	No difference in hand conductance.

**Patient or population:** patients with T2DM and neuropathic pain **Setting:** high-income countries (United States) (Kender et al., 2023) [58] **Intervention:** plant-based fasting-mimicking diet **Comparison:** standard care					
**Outcomes**	**Values Reported in Original Manuscript ^†^**		**№ of Participants** **(studies)**	**Certainty of Evidence** **(GRADE)**	**Comments**
	**Intervention Group**	**Control Group**			
NSS	Pre: 5.4 ± 0.8 Post: 4.1 ± 1.0	Pre: 5.5 ± 0.9 Post: 5.6 ± 0.9	31 (1 study)	⨁⨁◯◯ Low	Kender et al. (2023) did not report statistically significant findings.
Cold Detection Threshold	Pre: −1.13 ± 0.28 Post: −1.59 ± 0.25	Pre: −1.73 ± 0.35 Post: −1.96 ± 0.37	31 (1 study)	⨁⨁◯◯ Low	Kender et al. (2023) did not report statistically significant findings.
Warm Detection Threshold	Pre: −0.77 ± 0.23 Post: −0.95 ± 0.24	Pre: −1.37 ± 0.21 Post: −1.46 ± 0.30	31 (1 study)	⨁⨁◯◯ Low	Kender et al. (2023) did not report statistically significant findings.
Thermal Sensory Limen	Pre: −1.06 ± 0.20 Post: −1.00 ± 0.19	Pre: −1.57 ± 0.26 Post: −1.21 ± 0.30	31 (1 study)	⨁⨁◯◯ Low	Kender et al. (2023) did not report statistically significant findings.
Cold Pain Threshold	Pre: −0.45 ± 0.21 Post: −0.28 ± 0.20	Pre: −0.42 ± 0.23 Post: −0.46 ± 0.22	31 (1 study)	⨁⨁◯◯ Low	Kender et al. (2023) did not report statistically significant findings.
Heat Pain Threshold	Pre: −0.02 ± 0.36 Post: −0.47 ± 0.41	Pre: −0.76 ± 0.37 Post: −1.10 ± 0.30	31 (1 study)	⨁⨁◯◯ Low	Kender et al. (2023) reported a statistically significant difference within control group (*p* < 0.05).
Pain Pressure Threshold	Pre: 0.10 ± 0.29 Post: −0.09 ± 0.22	Pre: −0.38 ± 0.36 Post: −0.47 ± 0.41	31 (1 study)	⨁⨁◯◯ Low	Kender et al. (2023) did not report statistically significant findings.
Mechanical Pain Threshold	Pre: 1.81 ± 0.48 Post: 2.01 ± 0.55	Pre: 0.71 ± 0.66 Post: 0.36 ± 0.60	31 (1 study)	⨁⨁◯◯ Low	Kender et al. (2023) did not report statistically significant findings.
Mechanical Pain Sensitivity	Pre: 0.65 ± 0.30 Post: 0.77 ± 0.35	Pre: 0.18 ± 0.49 Post: 0.46 ± 0.52	31 (1 study)	⨁⨁◯◯ Low	Kender et al. (2023) did not report statistically significant findings.
Wind-up Ratio	Pre: −0.17 ± 0.23 Post: 0.04 ± 0.23	Pre: −0.16 ± 0.24 Post: 0.59 ± 0.53	31 (1 study)	⨁⨁◯◯ Low	Kender et al. (2023) did not report statistically significant findings.
Mechanical Detection Threshold	Pre: −0.30 ± 0.68 Post: −0.78 ± 0.41	Pre: −1.30 ± 0.64 Post: −1.52 ± 0.52	31 (1 study)	⨁⨁◯◯ Low	Kender et al. (2023) did not report statistically significant findings.
Vibration Detection Threshold	Pre: −1.70 ± 0.65 Post: −1.79 ± 0.62	Pre: −3.80 ± 0.79 Post: −1.91 ± 0.89	31 (1 study)	⨁⨁◯◯ Low	Kender et al. (2023) did not report statistically significant findings.
Peroneal Compound Muscle Action Potential (uV)	Pre: 5.50 ± 0.97 Post: 4.77 ± 1.09	Pre: 3.41 ± 0.79 Post: 3.61 ± 0.72	30 (1 study)	⨁⨁◯◯ Low	Kender et al. (2023) did not report statistically significant findings.
Peroneal Motor Nerve Conduction Velocity (m/s)	Pre: 38.65 ± 1.86 Post: 37.71 ± 1.94	Pre: 37.39 ± 2.28 Post: 37.08 ± 1.94	30 (1 study)	⨁⨁◯◯ Low	Kender et al. (2023) did not report statistically significant findings.
Sural Sensory Nerve Action Potential Amplitude (uV)	Pre: 3.96 ± 1.06 Post: 2.88 ± 0.69	Pre: 2.46 ± 0.64 Post: 2.10 ± 0.44	30 (1 study)	⨁⨁◯◯ Low	Kender et al. (2023) did not report statistically significant findings.
Sural Sensory Nerve Conduction Velocity (m/s)	Pre: 38.18 ± 1.81 Post: 37.12 ± 2.04	Pre: 38.54 ± 3.37 Post: 38.54 ± 2.46	30 (1 study)	⨁⨁◯◯ Low	Kender et al. (2023) did not report statistically significant findings.
Tibial Compound Muscle Action Potential (uV)	Pre: 7.79 ± 1.24 Post: 9.21 ± 1.45	Pre: 6.35 ± 1.47 Post: 6.38 ± 1.49	30 (1 study)	⨁⨁◯◯ Low	Kender et al. (2023) reported a statistically significant difference within intervention group (*p* < 0.05).
Tibial Motor Nerve Conduction Velocity (m/s)	Pre: 39.29 ± 1.55 Post: 36.29 ± 2.38	Pre: 37.23 ± 2.38 Post: 32.89 ± 3.05	30 (1 study)	⨁⨁◯◯ Low	Kender et al. (2023) reported a statistically significant difference within control group (*p* < 0.05).
Sciatic Nerve Fractional Anisotropy	Pre: 0.37 ± 0.02 Post: 0.40 ± 0.02	Pre: 0.37 ± 0.05 Post: 0.37 ± 0.04	13 (1 study)	⨁⨁◯◯ Low	Kender et al. (2023) did not report statistically significant findings.
Sciatic Nerve T2-Time	Pre: 72.85 ± 3.51 Post: 67.88 ± 3.35	Pre: 76.94 ± 7.17 Post: 75.21 ± 4.81	13 (1 study)	⨁⨁◯◯ Low	Kender et al. (2023) did not report statistically significant findings.

**Patient or population:** patients with chronic sciatica and neuropathic pain **Setting:** low-income countries (Iran) (Safari et al., 2020) [60] **Intervention:** low-calorie diet **Comparison:** standard care					
**Outcomes**	**Values Reported in Original Manuscript** **^†^**		**№ of Participants** **(Studies)**	**Certainty of Evidence** **(GRADE)**	**Comments**
	**Intervention Group**	**Control Group**			
MPQ-SF Sensory	Pre: 6.73 ± 1.41 Post: 4.46 ± 1.71	Pre: 5.77 ± 1.45 Post: 5.74 ± 2.11	96 (1 study)	⨁⨁◯◯ Low	Safari et al. (2020) reported a statistically significant difference within intervention group (*p* < 0.001) and between groups (*p* = 0.015).
MPQ-SF Affective	Pre: 0.98 ± 0.64 Post: 0.50 ± 0.62	Pre: 0.90 ± 0.63 Post: 0.87 ± 0.85	96 (1 study)	⨁⨁◯◯ Low	Safari et al. (2020) reported a statistically significant difference within intervention group (*p* = 0.002) and between groups (*p* = 0.002).
MPQ-SF Total	Pre: 7.71 ± 1.69 Post: 4.96 ± 2.02	Pre: 6.63 ± 1.44 Post: 6.62 ± 2.53	96 (1 study)	⨁⨁◯◯ Low	Safari et al. (2020) reported a statistically significant difference within intervention group (*p* < 0.001) and between groups (*p* = 0.001).
MPQ-SF PPI	Pre: 2.23 ± 0.47 Post: 2 ± 0.68	Pre: 2 ± 0.68 Post: 1.79 ± 1.3	96 (1 study)	⨁⨁◯◯ Low	Safari et al. (2020) reported a statistically significant difference within intervention group (*p* = 0.001), control group (*p* = 0.013), and between groups (*p* = 0.006).

**Patient or population:** patients with stage 3/4 chronic kidney disease and peripheral neuropathy **Setting:** high-income countries (Australia) (Arnold et al., 2017) [56] **Intervention:** potassium reduced diet (1 mmol/kg/day) **Comparison:** standard care					
**Outcomes**	Anticipated absolute effects *		**№ of Participants** **(studies)**	**Certainty of Evidence** **(GRADE)**	**Comments**
	**Risk with Standard Care**	**Risk with Diet (95% CI)**			
TNS	The mean change in TNS was 0	MD **2.4 lower** (−4, −30.8)	47 (1 study)	⨁⨁⨁⨁ High	Dietary lifestyle intervention improved neuropathy severity. Certainty upgraded due to large effect size.
Median Nerve Composite Excitability Score	The mean change in median nerve composite excitability score was 0	MD **7.4 higher** (5.96, 8.84)	47 (1 study)	⨁⨁⨁⨁ High	Dietary lifestyle intervention improved composite nerve excitability score. Certainty upgraded due to very large effect size.

**Outcomes**	**Values Reported in Original Manuscript ^†^**		**№ of Participants** **(Studies)**	**Certainty of Evidence** **(GRADE)**	**Comments**
	**Intervention Group**	**Control Group**			
SF36-Physical Function (median with IQR)	Pre: 75 (53–90) Post: 70 (40–80)	Pre: 60 (30–95) Post: 60 (26–94)	47 (1 study)	⨁⨁⨁◯ Moderate	Arnold et al. (2017) did not report statistically signfiicant findings.
Sural Sensory Nerve Action Potential Amplitude (uV)	Pre: 7.1 ± 10.5 Post: 6.2 ± 7.8	Pre: 8.9 ± 9.4 Post: 7.6 ± 9.3	47 (1 study)	⨁⨁⨁◯ Moderate	Arnold et al. (2017) did not report statistically signfiicant findings.

**Patient or population:** patients with gluten sensitivity and peripheral neuropathy **Setting:** high-income countries (United Kingdom) (Hadjivassiliou et al., 2006) [57] **Intervention:** gluten free diet **Comparison:** standard care					
**Outcomes**	Anticipated absolute effects * (95% CI)		**№ of Participants** **(Studies)**	**Certainty of Evidence** **(GRADE)**	**Comments**
	**Risk with Standard Care**	**Risk with Diet (95% CI)**			
Sural Sensory Nerve Action Potential Amplitude (uV)	The mean change in sural sensory nerve action potential amplitude (uV) was 0	MD **1.18 higher** (0.98, 1.38)	35 (1 study)	⨁⨁◯◯ Low	Dietary lifestyle intervention improved sural sensory nerve action potential amplitude.
Sural Sensory Nerve Conduction Velocity (m/s)	The mean change in sural sensory nerve conduction velocity (m/s) was 0	MD **2.26 higher** (−1.00, 3.52)	35 (1 study)	⨁⨁⨁◯ Moderate	No difference in sural sensory nerve conduction velocity. Certainty upgraded due to large effect size.

**Outcomes**	**Values Reported in Original Manuscript ^†^**		**№ of Participants** **(Studies)**	**Certainty of Evidence** **(GRADE)**	**Comments**
	**Intervention Group**	**Control Group**			
Subjective Neuropathy Perception	16/25 (64%) reported improvement	8/10 (80%) reported worsening	35 (1 study)	⨁⨁◯◯ Low	Hadjivassiliou et al. (2006) report patients in the control group were statistically significantly less likely to feel their neuropathy had improved (*p* < 0.0006).

**Patient or population:** patients with chronic lower back pain and neuropathic pain **Setting:** high-income countries (Turkey) (Torlak et al., 2020) [59] **Intervention:** intermittent high protein diet and mediterannean diet **Comparison:** standard care					
**Outcomes**	**Values Reported in Original Manuscript ^†^**		**№ of Participants** **(Studies)**	**Certainty of Evidence** **(GRADE)**	**Comments**
	**Diet + PT Group**	**PT Alone Group**			
LANSS	Pre: 10.6 ± 0.88 Post: 7.1 ± 0.76	Pre: 5.1 ± 0.42 Post: 2.6 ± 0.36	40 (1 study)	⨁⨁⨁◯ Moderate	Torlak et al. (2020) reported a statistically significant difference within diet + PT group (*p* < 0.001) and within PT alone group (*p* < 0.001).
VAS	Pre: 7.45 ± 0.44 Post: 4.7 ± 0.42	Pre: 6.65 ± 0.31 Post: 3.1 ± 0.59	40 (1 study)	⨁⨁⨁◯ Moderate	Torlak et al. (2020) reported a statistically significant difference within diet + PT group (*p* < 0.001) and within PT alone group (*p* < 0.001).

**GRADE Working Group grades of evidence. High certainty:** we are very confident that the true effect lies close to that of the estimate of the effect; **Moderate certainty:** we are moderately confident in the effect estimate: the true effect is likely to be close to the estimate of the effect, but there is a possibility that it is substantially different; **Low certainty:** our confidence in the effect estimate is limited: the true effect may be substantially different from the estimate of the effect; **Very low certainty:** we have very little confidence in the effect estimate: the true effect is likely to be substantially different from the estimate of effect.					

* **The risk in the intervention group** (and its 95% confidence interval) is based on the assumed risk in the comparison group and the **relative effect** of the intervention (and its 95% CI); **^†^ Difference in means not provided,** therefore anticipated absolute effects not calculated, and certainty of evidence not subject to change beyond baseline risk of bias; reported values are means, unless stated otherwise. **CI:** confidence interval; **IQR:** interquartile range; **LANSS:** Leeds assessment of neuropathic symptoms and signs; **mcg:** microgram; **MD:** mean difference; **mmol:** millimole; **MNSI-PA:** Michigan neuropathy screening instrument physical assessment; **MNSI-Q:** Michigan neuropathy screening instrument questionnaire; **MPQ-SF:** McGill pain questionnaire short form; **m/s:** meters per second; **NSS:** neuropathy symptom score; **NTSS:** neuropathy total symptom score; **Post:** value after interventional period; **Pre:** value prior to interventional period; **PT:** physical therapy; **SF36:** short form-36 health survey; **T2DM:** type 2 diabetes mellitus; **TNS:** total neuropathy score; **uS:** microsiemens; **uV:** microvolt; **VAS:** visual analog scale.

## Supplementary Materials

The following supporting information can be downloaded at: https://www.mdpi.com/article/10.3390/jcm13226766/s1, Table S1: Study exclusions; Table S2: Outcomes matrix for included studies.

## Glossary

BPI	Brief Pain Inventory
EORTC QLQ C-30	European Organization for Research and Treatment of Cancer Quality of Life Questionnaire Core-30
FACT-NTX	Functional Assessment of Cancer TherapyNeurotoxicity
GPS	Gracely Pain Scale
GRADE	Grading of Recommendations, Assessment, Development and Evaluation
LANSS	Leeds Assessment of Neuropathic Symptoms and Signs
MPQ	McGill Pain Questionnaire
MDNS	Michigan Diabetic Neuropathy Score
MNSI	Michigan Neuropathy Screening Instrument
NCV	nerve conduction velocity
NP	neuropathic pain
NPS	Neuropathic Pain Scale
NQOL	Neuropathy Quality of Life
NSS	Neuropathy Symptom Score
NTSS	Neuropathy Total Symptom Score
NPRS	Numeric Pain Rating Scale
PN	peripheral neuropathy
PPI	Present Pain Intensity
PSS	Pain Severity Scale
PNQ	Patient Neurotoxicity Questionnaire
QST	quantitative sensory testing
RCT	randomized controlled trial
ROB	risk of bias
SF36	Short Form-36 Health Survey
SPNS	Subjective Peripheral Neuropathy Screening
T2DM	Type 2 Diabetes Mellitus
mTCNS	modified Toronto Clinical Neuropathy Score
TNS	Total Neuropathy Score
VAS	Visual Analog Scale
